# Personal Circumstances Preceding Firearm Suicide Death Among Black Adults in the United States

**DOI:** 10.1007/s40615-024-02136-4

**Published:** 2024-09-09

**Authors:** Evan V. Goldstein

**Affiliations:** Department of Population Health Sciences, Spencer Fox Eccles School of Medicine, University of Utah, Williams Building Room 1N502, Salt Lake City, UT 84108 USA

**Keywords:** Black or African American, Self-injurious behavior, Suicide, Health disparities, Qualitative research

## Abstract

Despite an alarming rise in firearm suicide rates within Black communities, there has been little research on the contributors that precede firearm suicide deaths among Black adults. To fill this knowledge gap, this study was guided by an overarching research question: What are the recurring and salient personal circumstances experienced by Black adults prior to dying by firearm suicide in the US? This basic, interpretive qualitative study analyzed narrative text data for 843 Black adult firearm suicide decedents from the National Violent Death Reporting System. The analysis began with an initial cycle of open coding, followed by a second coding cycle, organizing the circumstances into major categories using a focused coding process. The analysis then moved back and forth between description and interpretation, and major themes were developed. Five themes emerged from this analysis: (1) Decedents often experienced poor health prior to death — but not always poor mental health. (2) Romantic relationships were often deteriorating, leading to frequent interpersonal arguments. (3) Alcohol and substance use were common before a suicide attempt. (4) Decedents frequently experienced financial and legal difficulties prior to death. (5) Decedents had many pathways to accessing firearms, and limiting firearm availability before death was challenging. Additional research on potential intervention pathways will be critical, given recent evidence indicating dramatic increases in firearm ownership among Black adults over the past 5 years.

**Supplementary Information:**

The online version contains supplementary material available at 10.1007/s40615-024-02136-4.

## Introduction

Suicide death has become more common among Black Americans over the past decade, with age-adjusted suicide rates among Black adults in the United States increasing by 36.6% from 2010 to 2020 (i.e., from 6.74 to 9.21 deaths per 100,000 persons) [1]. Firearm-related suicide deaths pose an especially concerning public health challenge among Black adults. In general, access to firearms — the most common and lethal method of suicide death [2, 3] — exacerbates suicide risk [4, 5]. Many people who attempt suicide ultimately survive [6]; however, survival is typically less likely for suicide attempts involving firearms, given the 80–90% case-fatality rate of firearm attempts [7, 8]. Firearm suicide rates among Black adults increased by 40.1% from 2010 to 2020 nationally (i.e., from 3.50 to 5.01 deaths per 100,000 persons) [1]. In 2022, firearms accounted for more than half of suicide deaths in the US overall [1] but proportionately more (58.7%) suicide deaths among Black adults [9]. The next most common mechanisms of suicide death among Black adults nationally were suffocation and drug-related poisoning, accounting for 19.8% and 7.1% of suicide deaths, respectively [9].

Systematic reviews of older studies have demonstrated that risks for suicide in general among Black adults (i.e., not just suicide deaths involving firearms) are likely influenced by sociocultural and healthcare system factors, such as acculturation [10], the loss of social support and family members [11], family conflict [12], being single and unmarried [11] or widowed [13], diminished religious well-being [14], economic strain [13], mental ill-health [14], limited access to population-appropriate mental health services [11], and distrust of mental healthcare providers [11]. Recent studies have demonstrated that depression [15] and pronounced feelings of hopelessness and a lack of accomplishment in life [16] are associated with suicidal thoughts and behaviors among Black young adults. However, despite the alarming rise in firearm suicide rates among Black adults in the US, there has been little research on firearm-specific suicide deaths within this population.

The high lethality of firearm suicide attempts poses significant but ambiguous prevention challenges. Previous studies have demonstrated that many individuals who have made serious suicide attempts thought about suicide for as little as 5–10 min before they acted [17], so safe firearm storage (or voluntary firearm removal) can make the difference between life and death among people at risk. Previous research has also suggested that when one method of suicide is not accessible, individuals rarely substitute different suicide methods [18]. Even if other suicide methods are substituted, the likelihood of death from the alternative methods will be reduced because firearms are the most lethal suicide method. According to theories of suicidal behavior aligned with the *Ideation-to-Action* framework, such as the *Interpersonal Theory of Suicide* and *3-Step Theory*, suicidal ideation can transition to suicidal behavior when one has the “capability” for death [19–21]. The capability for suicide is influenced by contributors that facilitate an individual to attempt suicide. In addition to *practical contributors*, such as those that increase knowledge of and access to firearms, *acquired contributors* can lower one’s fear of death through painful and provocative circumstances [20, 22]. Nevertheless, there has been little research on the contributors that precede firearm-related lethal suicide attempts among Black adults. Investigating these circumstances may be critical for saving lives within Black communities, especially given recent evidence indicating dramatic increases in firearm ownership among Black adults over the past 5 years [23–25].

To fill this knowledge gap, this study was guided by an overarching research question: What are the recurring and salient personal circumstances experienced by Black adults prior to dying by firearm suicide in the US?

## Methods

### Data

This study received de-identified data from the National Violent Death Reporting System (NVDRS) Restricted Access Database (RAD) [26] on all Black adult firearm suicide deaths from 2013 to 2021 through an approval process managed by the Centers for Disease Control and Prevention (CDC). The NVDRS program links data from vital records and investigative reports from coroners/medical examiners (CME) and law enforcement (LE) to compile comprehensive incident-level data on violent deaths from all 50 states. The NVDRS contains demographic, mortality, circumstance, and weapon information, as well as unstructured, free-text CME and LE narratives that the NVDRS program summarizes from interviews of persons close to each death. These records are linked to additional data sources, including toxicology and medical information, where available [27].

### Sample

The study sample included 843 adult firearm suicide decedents who died from 2013 to 2021. The decedents included in the study sample were aged 18 + at the time of death. Following a probability sampling approach, a 10% simple random sample of decedents was selected without replacement from all Black adult firearm suicide decedents included in the NVDRS over the study period. The representativeness of the study sample was assessed by testing the balance of eight covariates between the study sample and population using χ^2^ goodness of fit and *t* tests. As expected, given a population size of 8434 decedents, the 10% random sample closely mirrored the characteristics of the full population of available decedents, making it suitable for drawing inferences about Black adult firearm suicide decedents over the study period. The study sample was representative of all Black adult firearm suicide decedents in the US included in the NVDRS from 2013 to 2021 (i.e., by age, sex, ethnicity, educational status, marital status, military veteran status, death year, and geographically). The decedents were identified using the NVDRS program’s thorough process for determining suicide — which harmonizes manner of death designations from death certificates and CME investigations across many different jurisdictions — and standardized information on the weapon resulting in each death (i.e., firearm) [26, 27].

Not all states reported to NVDRS during the study period. Appendix Table A1 describes the states represented in the study sample. This study was deemed non-human subjects research using deidentified data on deceased persons only and did not require approval from the University of Utah Institutional Review Board. This study is part of a larger project that received an exemption determination by the University of Utah Institutional Review Board (IRB_00169397).

### Data Analysis

This was a basic, interpretive qualitative study [28] focused on describing the personal circumstances Black adults experienced in their interactions with their social worlds prior to firearm suicide death, as summarized through the CME and LE narratives. The free-text CME/LE narrative was the unit of analysis, and the CME/LE narratives were analyzed for the 843 decedents included in the study. Because the etiology of firearm suicide is understudied among Black adults, a predetermined list of circumstances was not used to code the narratives. Instead, the author immersed himself in the data and used an inductive process to review, code, and categorize recurring personal circumstances that preceded firearm suicide death as they were documented in the free-text data. This process allowed emergent themes to arise from the data itself. The analysis was conducted at a semantic level [29, 30] to enhance the credibility of the findings and stay as true as possible to the decedents’ experiences without extrapolating beyond the NVDRS abstractors’ reporting of each death.

The analysis began with an initial cycle of open coding, during which the data were broken down to identify personal circumstances preceding the firearm suicide deaths. Through this first coding cycle, circumstances were categorized using descriptive and in vivo codes [31]. The descriptive codes allowed for parsing, summarizing, and indexing circumstances as they occurred in the data, while the in vivo — or “verbatim” — codes allowed for indexing actual phrases and language found in the narratives, thus honoring the decedents’ voice in the analysis [31]. A codebook containing definitions (e.g., inclusion/exclusion criteria) and examples for each code was created at the beginning of the first coding cycle. New data were constantly compared with previously coded data throughout this cycle to identify similarities, differences, and emerging patterns [32]. Coding and analytic insights were documented through memos at the end of each coding session and whenever new insights emerged, new codes emerged, or existing codes were combined or redefined [33]. An audit trail was maintained to document the redefinition or combination of any similar codes throughout this process [29]. This process helped refine and expand the codebook throughout the first coding cycle.

The analysis continued with a second coding cycle, during which the circumstances were organized into major categories using a focused coding process [31, 34]. This process included five steps. First, two “code landscaping” techniques were used to visualize the descriptive and in vivo codes from the first coding cycle and build connections across the decedents included in the study [31]. One technique involved generating word clouds to inspect the commonality of the codes that emerged over the whole corpus of CME/LE data (i.e., more frequent circumstances represented by larger font sizes). Another technique involved mapping the co-occurrence of each first-cycle code with each other code in the data. These techniques provided insight into which codes were salient among the decedents, as well as initial commonalities and relations among the codes. Second, all analytic memos from the first coding cycle were reviewed. These memos included insights about categorical patterns in the data and any relationships that emerged among the circumstances present in the narratives. Third, the CME/LE narratives were re-reviewed across the decedents to assess the comparability of specific circumstances within the emergent categories. Fourth, to enhance credibility and trustworthiness, major circumstance categories were triangulated [28, 35] with previously coded NVDRS data — when possible, e.g., validating the occurrence of known mental health conditions — and insights from the academic literature. Fifth, alternative categorization schemes were brainstormed, and counterexamples [36] were explored with less commonly co-occurring first-cycle codes. Codes that were not cross-referenced enough to fit into larger categories were either further combined or eliminated from this analysis stage. Data were coded and organized using Dedoose Version 9.2.6.

Finally, once the major categories were coded and reflected upon, the analysis moved back and forth between description and interpretation, taking a reductionist approach to develop three to five major themes that best conveyed the author’s interpretations of the recurring and salient personal circumstances that precede firearm suicide death among Black adults in the US [30, 31, 37, 38].

### Thematic Case Examples Using Composite Narratives

The NVDRS RAD is a de-identified, case-level dataset. The data are stored without personally identifiable information in a secure national database maintained by CDC. That said, incident narrative text may hypothetically identify a decedent in the instance of deaths that were high-profile or well-known to a community or individuals familiar with the decedent. Following guidelines provided by the NVDRS program and to further protect the identities of the decedents included in this analysis, any example from the CME/LE narratives shown below has been anonymized through a process that omits/reconstructs specific details and combines salient information from multiple decedents that share circumstances that overlap thematically in the form of a composite narrative. Furthermore, gender-neutral descriptions have been used for any decedents described in the composite narratives. No individual narrative is referenced directly on its own.

### Researcher Reflexivity and Trustworthiness

In addition to triangulation and providing a thick description of the analytic procedures and findings [39], the author strove to mitigate his biases and deepen the analysis’ trustworthiness by acknowledging how his identity and experiences may have affected his interpretation of the CME/LE data. Although this study was not guided by critical theory, nor was there any interaction with human subjects, the author remained highly attentive to the power relations inherent in this work. Understanding an author’s positionality is crucial for determining the credibility of an interpretive analysis of qualitative data [40]. Positionality is the “relational place or value one has that influences and is influenced by varying contexts (e.g., social, political, historical, educational, and economical to name a few)” and roles [41]. This positionality statement helps uncover the author’s subjectivities in the analytic process; it sheds light on how the author interpreted the CME/LE narratives and abstracted meaning from the likely subjective truths experienced by the decedents prior to death. This statement provides a glimpse into why and how the author made his interpretations.

The author’s interpretive lens has developed over time. The author is a middle-class white male of Central and Eastern European descent. The author was raised in a split nuclear family in a lower-middle-class inner-ring suburb of Cleveland, Ohio. Although the author’s family espoused a “color-blind” racial ideology during his upbringing, the author began the process of learning the subtleties of racialization in the US as an undergraduate student in New Orleans, LA, before studying the influences of racism on Black wellness, the Public Health Critical Race Praxis [42], theories of justice, and the social construction of identity and knowledge as a graduate student and academic. Before becoming an academic, the author served in various community health roles in predominantly Black communities experiencing significant health disparities and social inequities in Cleveland. It was through these experiences that the author first observed and reflected on the impact of social circumstances and racialization on health.

Despite the author’s commitment to investigating health disparities through an equity lens, the author is an “outsider” [41] to the community he is studying and can never fully understand the experiences of the Black decedents in this study, nor fully understand how their racialized identities may have affected the events leading up to their deaths. Epistemologically, the author identifies as a constructionist and believes that knowledge is socially created, partial, and dependent on context and interpretation. The author is thus aware of how an analysis such as this has the potential to perpetuate racial hierarchies in the production of new knowledge [42]. For these reasons, in this study, the author was deeply committed to generating credible descriptive insights in response to the research question stated earlier. He did so by elevating and describing the experiences of Black adult firearm suicide decedents without extrapolating beyond the details included in the CME/LE narratives or making comparisons to other populations. Because previous studies have demonstrated racial/ethnic differences in the length and choice of language used in the CME/LE narratives, the author also acknowledges that the data being analyzed itself may contain biases in whether certain information was included for Black decedents by the NVDRS program staff who abstracted the CME/LE narratives, e.g., information about social disadvantages or the effects of structural racism may not have been reported or may have been inadvertently misrepresented by LE, CMEs, or the NVDRS. As a middle-class white man in academia, the author was in a privileged position to undertake this task with federal research funding support. Throughout this study, the author examined his influence in this process through memo writing and constant self-reflection [33].

## Findings

Table 1 describes the study sample. The decedents had an average age of 38.2 years. The majority of the decedents were male (88.6%) and identified as non-Hispanic/Latino/a (97.8%). About 45.0% of the decedents were high school graduates or completed their GED, while about 9.3% had a bachelor’s, master’s, doctorate, or professional degree. Nearly one in four (22.8%) of the decedents were married or in a civil union or domestic partnership at the time of death. About 16.9% of the sample served in the US Armed Forces. As shown in Appendix Table 1, the study sample was representative of all US Black adult firearm suicide decedents documented in the NVDRS from 2013 to 2021.

**Table 1:** Describing the study sample (*n* = 843), 2013–2021

	Mean/frequency	Standard deviation/percent
Age in years, mean, SD	38.2	16.4
Sex, *n*, %		
Male	747	88.6%
Female	96	11.4%
Ethnicity, *n*, %		
Not Hispanic/Latino/a	823	97.6%
Hispanic/Latino/a or Unknown	20	2.4%
Education level, *n*, %		
8th grade or less	12	1.4%
9th to 12th grade, no diploma	111	13.1%
High school graduate or GED completed	379	45.0%
Some college credit, but no degree	167	19.8%
Associate’s degree	58	6.9%
Bachelor’s degree	55	6.5%
Master’s, doctorate or professional degree	23	2.8%
Unknown	36	4.5%
Marital status, *n*, %		
Married/civil union/domestic partnership	192	22.8%
Never Married	483	57.3%
Widowed	29	3.4%
Divorced	90	10.7%
Married/civil union/domestic partnership, but separated	21	2.5%
Single, not otherwise specified	17	2.0%
Unknown	11	1.3%
Military veteran status, *n*, %		
Did not serve	684	81.1%
Served in the US armed forces	142	16.9%
Unknown	17	2.0%
Death year, *n*, %		
2013	36	4.2%
2014	39	4.6%
2015	49	5.8%
2016	80	9.4%
2017	83	9.9%
2018	117	13.9%
2019	108	12.8%
2020	144	17.1%
2021	187	22.3%
Census region		
West	77	9.1%
Midwest	221	26.2%
Northeast	78	9.3%
South	467	55.4%

Author’s analysis of National Violent Death Reporting System (NVDRS) Restricted Access Database (RAD) data

The CME/LE narratives had a combined average length of 225 words (standard deviation = 156 words) per decedent. Appendix Table A3 provides a list of 72 first-cycle codes. Nine major categories were ultimately organized through the second coding cycle: Health, Interpersonal Conflicts, Substance Use, Financial Issues, Legal Issues, Firearm Access, Firearm Restriction, Seeking Escape, and People at the Incident. The analysis of the recurring and salient personal circumstances that precede firearm suicide death among Black adults in the US revealed five major themes. Each theme encompasses salient personal circumstances that appeared to precipitate or facilitate a firearm suicide attempt. The themes are discussed and illustrated with composite textual examples from the CME/LE narratives constructed from multiple decedents sharing circumstances that overlap thematically.

### Theme 1: Decedents Often Experienced Poor Health Prior to Death — But Not Always Poor Mental Health

Health-related issues were definitive circumstances preceding lethal firearm suicide attempts in this study. The range of health conditions included both mental and physical illnesses and diseases, though mental health disorders were most common (i.e., about 29.0% of the decedents). Clinically diagnosed depressive and anxiety disorders were the most recurring, as were previous suicidal ideation and attempts. Serious mental illnesses, such as bipolar disorder and schizophrenia, were also present, as were many occurrences of decedents feeling depressed or stressed without a clinical diagnosis. Notably, nearly 20.7% of the decedents who experienced a known mental health disorder had stopped treatment for their conditions prior to death. Treatment cessation included no longer taking prescribed psychopharmacologic medications or attending psychotherapy. Stopping treatment often appeared to be the decedent’s decision; however, there was little information in the CME/LE narratives about provider, insurance, or other financial-related barriers to care. In some instances, prescribed medications were stopped and replaced with self-medication using alcohol or other recreational/illicit substances. On more than one occasion, family members and romantic partners expressed concerns when decedents refused to take their medications, as well as confusion and skepticism about the efficacy of certain treatments. Family members also worked hard to encourage decedents to continue their treatment up until the fatal suicide attempts, e.g.:

The decedent had a history of post-traumatic stress disorder. The decedent was taking several mental health/mood-related medications but had stopped taking their prescription medications several times over the years. The decedent received a new prescription medication a few weeks prior, but they said it made them feel worse. The family had confronted the decedent several times about reengaging in counseling. The decedent had recently agreed to see a mental health provider again.

Poor health circumstances were not exclusively mental health–related. Physical ill-health, pain, and disability were also recurring (about 16.5% of decedents). Terminal illnesses, potentially life-threatening conditions, and physical disabilities were recurring, including cancer, sickle cell disease, heart failure, amputation, blindness, paralysis, Parkinson’s disease, and multiple sclerosis, which often caused considerable pain and suffering prior to death. The amount of suffering caused by physical ill-health was often too great to endure, as exemplified by the following composite narrative:

The decedent had been diagnosed with colon cancer several years ago but had been successfully treated. However, several days prior to their death, the decedent told their spouse that they had recently been diagnosed with a new cancer-like growth. The decedent was scheduled to return to the hospital a week after their death for additional tests to receive more information about the growth. The decedent had survived a previous suicide attempt involving prescription medications, but the thought of having cancer again overwhelmed the decedent.

More commonly, decedents experienced chronic health conditions, such as diabetes mellitus, hypertension, chronic obstructive pulmonary disease, gastrointestinal disorders, vertigo-related disorders, and chronic kidney disease, and symptoms like vomiting and diarrhea, as well as pain, including low back pain, sciatic nerve pain, and neuropathy. As illustrated by the following composite narrative, physical ailments frequently deteriorated a decedent’s willingness to live and accelerated the capability of succumbing to death:

The decedent had been in and out of the hospital for the past several weeks due to liver disease. The decedent had been in a quiet mood leading up to their death, reportedly could not take the pain, stomach issues, and vomiting. The decedent’s quality of life was noticeably deteriorating. In a note to the decedent’s family, the decedent apologized and asked them for forgiveness.

### Theme 2: Romantic Relationships Were Often Deteriorating, Leading to Frequent Interpersonal Arguments

Many decedents got into arguments shortly before taking their own lives. These arguments were typically intense and passionate between decedents and their romantic partners. Romantic relationship troubles occurred for decedents of all ages and relationship types, including long-standing marriages, recent engagements, and individuals who only recently began to date each other. Infidelity — by either the decedent or the partner — was often a catalyst for transitioning from suicidal ideation to suicidal behavior, particularly when decedents appeared to learn about their partner’s infidelity unexpectedly, e.g., from a partner’s text messages or catching a partner leaving a club with another person. Illustrative examples suggested that infidelity caused either intense anger or sorrow and grief among the decedents. When infidelity begot anger, seemingly impulsive behaviors ensued, such as the use of firearms against their partners or other people before taking their own lives. There were also repeated examples of individuals dying by firearm suicide after committing domestic violence or being abused (both women and men). As illustrated by the following composite narrative, when infidelity led to sorrow and grief, decedents frequently turned to alcohol or recreational/illicit substances before seeming to lose the will to live:

The decedent’s spouse told the victim that they had been cheating on the victim again for weeks. The two began arguing in their backyard, and the decedent’s spouse told him that they had to separate for good. The spouse left, and the decedent was later found deceased in their apartment by the spouse’s sister. The decedent’s apartment was in disarray. Marijuana and empty beer cans were present. A note left for the spouse indicated that the decedent could not live without the spouse.

There were also numerous counterexamples where infidelity did not occur. In these instances, ongoing arguments with romantic partners and the gradual deterioration of romantic relationships appeared to exacerbate — or be exacerbated by — decedents’ underlying life challenges and precipitate firearm suicide attempts. These challenges included financial struggles, coping with the loss of loved ones, child custody disputes, and the combination of other life stressors, as illustrated by the following composite narrative:

The decedent and their spouse had been having problems. They were arguing a lot but continued to live together. The decedent was stressed about their relationship problems and working multiple jobs to pay child support. The decedent did not have money to pay rent but did not want to break up and lose the children. On the day of the incident, they were arguing again. The spouse told the decedent that maybe they should break off their relationship. The decedent became very upset, and brandished their handgun. Prior to death, the decedent ruminated further on the stresses of their life, including missing the decedent’s late family member. The decedent expressed remorse for their relationship troubles and love for the spouse.

### Theme 3: Alcohol and Substance Use Were Common Before a Suicide Attempt

About one in four (24.6%) of Black adult firearm suicide deaths involved alcohol or other recreational/illicit substances. Substance use was reported through the CME/LE investigative reporting process through interviews of next-of-kin, observations from law enforcement or coroner/medical examiners, and supplemental toxicology information included in the CME/LE narratives by the NVDRS program. In order of frequency, alcohol and cannabis were most commonly used on the day of death. Substances such as cocaine (and levamisole-adulterated variants), MDMA, delta-9-tetrahydrocannabinol, K2, methamphetamine, and non-prescribed medications such as benzodiazepines and opioids were also used repeatedly prior to death. Consistent with the previous two themes, for many decedents, recreational/illicit substances appeared to be used to help cope with the underlying stresses of life — most frequently for coping with mental ill-health and the deterioration of romantic relationships. Substance use was also recurring following intense arguments with romantic partners and among decedents with known histories of suicidal ideation, e.g.:

The decedent and their romantic partner were having an argument. Because of the argument, the romantic partner was leaving the apartment with her child. The romantic partner brough her child to her vehicle and went back into the residence where she found the decedent in the bedroom unresponsive. The decedent struggled with depression. The decedent left a voicemail for the romantic partner the morning of their death talking about their long struggle with depression and suicidal ideation from an early age. The decedent had been self-medicating per the decedent’s family with alcohol and other drugs.

Although not as frequently documented in the CME/LE narratives, 53 decedents had a known history of substance use disorder. Among those decedents, arguments on the day of death appeared to be less common than among the decedents who consumed alcohol or other recreational/illicit substances on the day of death. Co-occurring mental health disorders were more frequent among the decedents with known substance use disorders.

There were also counterexamples suggesting substance use may not have contributed to precipitating a suicide attempt — and that alcohol and other substances may not have been used as a coping mechanism. For example, there were decedents who happened to be having a beer with coworkers prior to death, and there were toxicology reports noting relatively low blood alcohol levels of 0.08 to 0.10.

### Theme 4: Decedents Frequently Experienced Financial and Legal Difficulties Prior to Death

Financial and legal problems often preceded firearm suicide deaths in this study. The range of financial issues included recently losing a job or pension, the necessity of working multiple jobs and long hours to support a family, the inability to afford rent or mortgage payments and property taxes, being evicted from an apartment or experiencing a home foreclosure due a lack of financial resources, experiencing homelessness or living in low-quality public housing, vehicles being repossessed, owing money to the Internal Revenue Service or other people, and incurring large gambling debts. There were even instances of older decedents who, overwhelmed by the financial burden of caring for multiple family members without incomes, took their own lives so that their family members would receive life insurance death benefits.

Financial difficulties often appeared to be catalysts precipitating a suicide attempt, especially new and traumatic experiences like homelessness. As illustrated by the following composite narrative, employment-related challenges were particularly harmful:

The decedent had lost their job due to the pandemic, and their car was recently repossessed. The decedent had a job interview recently before their death and could have been depressed because they did not get the job. The decedent and their romantic partner were having financial troubles, consisting of credit card debt and back taxes. A note with debts was also found. The victim recently stated that they didn’t know how they were going to survive financially.

Such experiences may have been characteristic of deeper socioeconomic disadvantages and fears (e.g., of losing one’s home) and exacerbated co-occurring emotional strain and mental ill-health. Multiple CME/LE narratives specifically mentioned decedents’ feeling despondent due to unobtainable career aspirations and not being able to achieve the things they had dreamed of at a younger age. However, deeper structural disadvantages were infrequently noted and difficult to infer from the CME/LE narratives.

Consistent with the previous themes, financial difficulties frequently co-occurred with ill health, relationship issues, substance use, and other stressors. Employment and financial difficulties were linked to legal issues experienced by numerous decedents. In some instances, legal issues such as previous arrests and court hearings for sexual misconduct directly affected the decedents’ employment. In other occurrences, employment and financial troubles appeared to cause or worsen the decedents’ legal circumstances, as illustrated in the following compositive narrative:

The decedent and spouse were having financial difficulties and recently received an eviction notice from their landlord. The decedent did not have regular employment and was dealing with a few legal problems recently. The decedent relied on cash from a check cashing scheme and shoplifting. The decedent was also recently charged with driving under the influence prior to death.

Decedents also frequently experienced legal issues and even criminal histories without financial difficulties. Legal issues often weighed heavily on these decedents, seemingly compounding and exacerbating the stresses of mental ill-health, romantic relationship issues, and other circumstances. Recurring counterexamples, however, illustrated decedents involved in serious and violent crimes, including suspected murder, rape, sexual assault, physical assault, pedophilia, and drug trafficking, prior to taking their own lives. For these decedents, access to firearms often proved to be harmful not only to themselves but also to others. Among these decedents, firearm violence against others — especially firearm violence against female romantic partners perpetrated by male decedents — was not uncommon prior to death.

### Theme 5: Decedents Had Many Pathways to Accessing Firearms, and Limiting Firearm Availability Before Death Was Challenging

Information on the type of firearms used in the lethal suicide attempts was not always available in the CME/LE narratives, though, when it was, a range of firearms was found to be accessible to the decedents. Consistent with previous studies [43], semi-automatic handguns were most commonly used. The handguns were chambered in various sizes, but centerfire 9 × 19 mm Parabellum pistols were the single most recurring type (i.e., about 17.6% of decedents). Other cartridge sizes that the handguns were chambered for included 0.40 Smith & Wesson, 0.380 ACP, 0.38 Special, 0.45 ACP, 0.357 Magnum, 0.32 ACP, and 0.22 LR. Shotguns and rifles were also involved in numerous suicide attempts, though less commonly than handguns.

On numerous occasions, decedents recently purchased the firearms they used to take their lives. Firearms were acquired both legally and illegally (e.g., unlicensed and straw purchases bypassing required background checks), sometimes within 24 h of a suicide attempt, e.g.:

The day before the fatal injury, the decedent went out and legally bought the gun and kept it in the room unlocked.

Just as frequently, decedents borrowed or stole firearms from parents, family members, romantic partners, and friends. On multiple occasions, decedents were able to access firearms due to their military veteran status or employment as law enforcement or security personnel. Having multiple firearms was a recurring circumstance.

Firearms were often unsafely stored and unlocked in the home, though there were numerous instances of firearms that were safely locked and stored prior to death (e.g., trigger locks and lock boxes). For some decedents, firearms were actively carried. Although not frequently documented in the CME/LE narratives, there were recurring instances of firearms being removed from the home because a decedent’s family recognized the importance of creating space and time between lethal means and an individual experiencing suicidal ideation. However, there were also instances of unsuccessful attempts to remove firearms from suicidal decedents’ homes, as well as several instances of firearms being recently returned to the decedents. The reasons for not being able to separate a decedent from their firearm during a crisis were interpersonally complicated. Reasons included a decedent’s desire to keep their firearm to protect themselves or maintain their right to bear arms and family members’ reluctance to intervene even when it was known that a decedent had little knowledge of safe firearm behavior, e.g.:

The decedent had previously attempted suicide several months prior. The decedent’s spouse asked the decedent’s mother to take away the decedent’s gun, but she refused to have that conversation because the decedent wanted to keep it for protection. The decedent retained their firearm despite not being known to have any significant training or other knowledge of firearm safety.

## Discussion

This study inductively examined recurring and salient personal circumstances experienced by Black adults prior to dying by firearm suicide in the US. Five major themes emerged from this analysis: (1) Decedents often experienced poor health prior to death — but not always poor mental health. (2) Romantic relationships were often deteriorating, leading to frequent interpersonal arguments. (3) Alcohol and substance use were common before a suicide attempt. (4) Decedents frequently experienced financial and legal difficulties prior to death. (5) Decedents had many pathways to accessing firearms, and limiting firearm availability before death was challenging. Each of these themes captured prominent personal circumstances preceding lethal firearm suicide attempts among Black adults from 2013 to 2021. For many decedents, salient personal circumstances were often co-occurring. This overlap was expected given that suicide is a complex and multifaceted phenomenon influenced by bio-psychological (e.g., feelings of not belonging socially and perceptions of being a burden), social, and economic factors [21, 44]. These findings are insightful given the dearth of research on firearm suicide death in Black communities and recent evidence indicating dramatic increases in firearm ownership among Black adults over the past 5 years [23–25]. Consistent with the *Interpersonal Theory of Suicide* and *Ideation-to-Action* framework, the themes that emerged from this study provide recent evidence about the personal “contributors” [22] that influence firearm suicide attempts among Black adults.

Data triangulated from other sources support the main findings. First, mental and physical ill-health often precipitated lethal suicide attempts among Black adults in this study. Consistent with the frequency of mental ill-health recurring among the decedents in this study, about 26.7% of the decedents had a known mental health or psychiatric problem documented in the pre-coded NVDRS data. Moreover, nearly 36.6% of the decedents who experienced poor physical health also experienced a known mental health disorder, which is consistent with older studies demonstrating the co-occurrence of poor physical and mental health among adult suicide decedents [45]. Second, older studies analyzing quantitative data have also found that acute alcohol use has been common prior to firearm suicide death among Black adults ages 18 + , [46] and the use of other recreational/illicit substances has been associated with suicide versus natural death among Black decedents ages 15–64 [47]. The relationship between acute alcohol/substance use and suicide attempts may relate to the neurobiological effects of alcohol and other substances on rapid, unplanned (i.e., impulsive) behavior and disinhibition, though the relationships among substance use, impulsivity, and suicidality are complicated [48–53]. Third, for generations, authors including W.E.B. Du Bois and Ida B. Wells have discussed how Black Americans have experienced intense socioeconomic disadvantages and the compounding of multiple, interpersonal, and structural social disadvantages [54, 55], leading to hopelessness and social isolation. Such writings appear to reflect the experiences of decedents in this study who underwent financial, legal, and other socioeconomic difficulties prior to death, often in combination with declining health and interpersonal relationships.

To that end, it is important to note that structural and systemic inequities may underlie or exacerbate the salient personal circumstances preceding firearm suicide death among Black adults. A legacy of structural racism helped shaped American society, impacting social order, privilege, and power differentials across groups of people. For individuals with racialized identities, especially Black identities, structural inequities created cumulative economic and political disadvantages that have persisted across generations, making it more challenging for Black individuals to access opportunities and resources [42]. The cumulative stresses and traumas from these generational disadvantages have negative health consequences within Black communities, likely including higher risks for mental ill-health and suicidality [56–58].

The main findings reported in this paper have important implications for future prevention pathways. Specifically, for individuals experiencing health-related concerns, previous studies have suggested that lethal means counseling may help reduce firearm suicide attempts and improve opportunities to treat underlying health needs [59–62]. One such intervention — Counseling on Access to Lethal Means (CALM) — incorporates counseling strategies for health professionals to help individuals and their families limit access to firearms and other lethal means in times of crisis, including dialogue on safe and secure firearm storage options [63]. CALM can be delivered alone or as part of a broader Safety Planning intervention [64]. The decision to administer CALM often depends on whether a patient shows signs of mental illness, substance use, or suicidal thoughts — but not physical health conditions or deteriorating socioeconomic circumstances. Consequently, this study may indicate opportunities to broaden the criteria for initiating CALM in healthcare settings with Black adults who experience not only mental illness or suicidal ideation but also chronic physical conditions, recent romantic relationship troubles, unemployment, or legal difficulties. A criteria expansion may help communities address *Goal #3* of the *2024 National Strategy for Suicide Prevention,* which aims to reduce access to lethal means among people at risk of suicide in the US broadly [65].

Nonetheless, additional research is needed to better understand how lethal means counseling may best serve Black adults. Previous studies evaluating lethal means counseling effectiveness or implementation often have not included racially/ethnically diverse individuals or communicated findings centered on the experiences of Black adults. As such, other researchers have underscored the need for incorporating racially/ethnically diverse and representative perspectives on lethal means counseling in subsequent studies [66]. In addition to known barriers to discussing mental health issues or accessing mental health care, there is little systematic knowledge about the barriers to lethal means safety among Black adults. In this study, there were notable instances of unsuccessful attempts to limit access to firearms among Black adults prior to death; in other instances, firearms had been recently returned to decedents under the premise that the risk for suicidal behavior had passed. Complicating matters, the decedents in this study accessed a wide array of firearm types, and the firearms were purchased both legally and illegally and even taken from romantic partners, family members, and friends. Ubiquitous firearm accessibility further underscores the importance of interventions like CALM and broader firearm safety practices. Several studies have demonstrated that Black adults are willing to talk to physicians and medical professionals about firearm safety [67, 68], though family members, military veterans, and law enforcement officers are generally viewed as more favorable firearm safety messengers [68]. Thus, the discussion of safe firearm storage or temporary firearm removal by family members, romantic partners, and peers of at-risk Black adults may be more effective than relying only on such conversations being had in healthcare settings. Faith-based intervention and community-based settings may also help facilitate CALM with Black adults, as faith- and community-based mental health interventions have been found to improve Black adults’ attitudes about mental health more generally [69].

Any conversation about firearm removal within Black communities must consider the Black historical tradition of arms as chronicled by Johnson (2014) [55]. For generations, many Black Americans have deeply valued their individual right and choice to own firearms as a means of self-defense and self-preservation, stemming from generations of facing overt and violent racism in the US [55]. Notably, “gun culture” in the US is not monolithic. There are many types of firearm owners and reasons for owning firearms [70, 71], such as the need for self-protection, recreation/hunting, and rights/activism. Moreover, many Americans are vehemently opposed to firearms, including many Black Americans [72]. Yet, the right to keep and bear arms is constitutionally protected in the US. Today, that right is conferred regardless of race or ethnicity, though that was not always so. To ask Black Americans who favor their firearms or keep their firearms for self-protection to relinquish their firearms — even if temporarily and for their own protection — may be an affront to their deeply-held rights, values, and identity. Nevertheless, when temporary firearm removal is not an option, helping a loved one improve their firearm storage practices in times of crisis can be impactful. One example would be helping an at-risk peer complete an online decision aid such as *Lock to Live*, which can help convey the relationship between firearm access and suicide death, provide information about the cost of different safe firearm storage options, plan for safe storage, and identify others who can help facilitate their plan [73]. Even among adults who are ultimately not at risk for suicide, lethal means counseling and the provision of firearm locks may improve safe firearm storage practices [74].

Additionally, consistent with *Goal #2* of the *2024 National Strategy for Suicide Prevention*, upstream interventions will be critical for preventing firearm suicide death in Black communities. In particular, public policymakers have opportunities to address the structural inequities exacerbating the salient personal circumstance preceding firearm suicide death among Black adults. Such initiatives may include fiscal policies that address socioeconomic disadvantages experienced by predominantly Black communities, as well as initiatives that reduce mental health stigma among Black adults [75] and expand mental health treatment capacity in Black communities, e.g., reducing long lag times between implementing policy solutions for enhancing training programs and growing the mental health workforce [76]. This is especially important for Black Americans who may have access to firearms, as greater mental health treatment capacity is associated with lower firearm suicide rates [77].

Finally, it is worth noting that salient personal circumstances preceding firearm suicide death even among Black adults only may vary by subpopulation, even though many risk factors are commonly applied across populations and over time [78]. For example, prior studies have demonstrated clinical differences between male and female suicide decedents (i.e., somatic symptomology, mental health diagnosis, and impulsivity differences) [79–81], though much less is known about social and economic circumstances preceding the death of women by firearm suicide [82], especially Black women. Moreover, about 16.9% of the decedents in this study served in the US Armed Forces. Military veterans experience elevated suicide risk [83] and are often exposed to factors linked to suicide, such as post-traumatic stress disorder and access to lethal means [83–85]. Multiple decedents in this study had easy access to firearms while experiencing suicidal ideation because of their military veteran status. Nevertheless, this study took a crucial step by analyzing information from a large sample of decedents representative of all Black adult firearm suicide decedents in the NVDRS over a long timeframe for the purpose of better understanding personal circumstances experienced by Black adults prior to firearm suicide death.

### Limitations

This study had several limitations. First, this analysis was not causal. Identifying causal links between various risk factors and firearm suicide death was not the intent. Rather, this study sought to describe and interpret recurring and salient personal circumstances that precede firearm suicide death among Black adults in the US. In addition to filling a critical research gap, the themes that emerged from this analysis will help inform additional studies investigating intervention pathways. Second, the NVDRS program synthesizes the narrative data from CME/LE investigative reports, incorporating information primarily derived from interviews with the decedents’ next-of-kin. This process may cause several issues with what information was reported through the CME/LE narratives. Next-of-kin interviews may be subject to recall bias or different types of informants may provide more or less salient circumstance information depending on their relationship and proximity to the decedent. Moreover, suicide can be a taboo topic and previous evidence has shown that Black Americans can be reluctant to acknowledge mental health issues or unable to access mental health care [86]. Thus, it is also possible that the decedents’ next-of-kin may have been unaware of specific but salient circumstances experienced by the decedents before death. That said, NVDRS abstractors commonly have the opportunity to incorporate information from other documents (e.g., toxicology and medical data) into the CME/LE narratives, whether included with or in addition to the investigation reports. To that end, it is possible that certain information may have eluded the NVDRS abstraction process. Third, previous studies have demonstrated racial/ethnic differences in the length and choice of language used in the CME/LE narratives [87]. Although original informant statements are not available through NVDRS, nor are the CMEs’ original reports, these differences may create biases in whether certain information was included for Black decedents by the NVDRS program staff who abstracted the CME/LE narratives, e.g., information about social disadvantages or the effects of structural racism may not have been reported or may have been misrepresented in the data. Fourth, in general, suicide death may be underreported due to limited information, the attentiveness of CMEs, or the misclassification of the cause of death on Black adults’ death records. To help address these concerns, the NVDRS program uses multiple data sources to improve demographic classification; moreover, the intent of self-induced death by firearm is often expository (i.e., determining whether the death was intentional versus accidental or undetermined). However, it is possible that Black adult firearm suicide decedents may have been excluded from this analysis; the circumstances experienced by such decedents may have differed from the circumstances experienced by the decedents included in the study. Fifth, this study was interpretive, the author’s own biases and perspectives may have influenced the analysis. In an effort to improve the credibility of the findings and further overcome the limitations of this study, the author described his positionality, described the analytic process in detail, and triangulated the main findings with other forms of available NVDRS data (e.g., pre-coded data on mental health status), the existing scholarly literature, and historical accountings (e.g., Well’s views on socioeconomic disadvantage), where possible.

## Conclusions

This study characterized recurring and salient personal circumstances experienced by Black adults prior to dying by firearm suicide in the US. The study sample was large and representative of all US Black adult firearm suicide decedents documented in the NVDRS RAD from 2013–2021. Five major themes emerged from this analysis: (1) Decedents often experienced poor health prior to death — but not always poor mental health. (2) Romantic relationships were often deteriorating, leading to frequent interpersonal arguments. (3) Alcohol and substance use were common before a suicide attempt. (4) Decedents frequently experienced financial and legal difficulties prior to death. (5) Decedents had many pathways to accessing firearms, and limiting firearm availability before death was challenging. Additional research on potential intervention pathways will be critical, especially given recent evidence indicating dramatic increases in firearm ownership among Black adults over the past 5 years.

## Supplementary Information

Below is the link to the electronic supplementary material.

Supplementary file1 (DOCX 26.9 KB)

## Data Availability

Per the author’s data use agreement with the Centers for Disease Control and Prevention, the raw NVDRS RAD data used for this study will be destroyed within 3 years of the start of the study. The NVDRS Restricted Access Database data are obtainable through an approval process with the CDC: cdc.gov/violenceprevention/datasources/nvdrs/dataaccess.html.
