# Supplementary material for: Personal Circumstances Preceding Firearm Suicide Death Among Black Adults in the United States
Source: J Racial Ethn Health Disparities. 2024 Sep 9;12(5):3339–52. doi: 10.1007/s40615-024-02136-4 (PMC12446094; doi:10.1007/s40615-024-02136-4)
Supplement: Supplementary file 1 — Supplementary file1 (DOCX 26.9 KB) [file 40615_2024_2136_MOESM1_ESM.docx]

Appendix Table A1. States represented in the study.

| **State** |
| --- |
| Alabama |
| Alaska |
| Arizona |
| Arkansas |
| California |
| Colorado |
| Connecticut |
| Delaware |
| District of Columbia |
| Georgia |
| Hawaii |
| Illinois |
| Indiana |
| Iowa |
| Kansas |
| Kentucky |
| Louisiana |
| Maine |
| Maryland |
| Massachusetts |
| Michigan |
| Minnesota |
| Mississippi |
| Missouri |
| Nebraska |
| Nevada |
| New Hampshire |
| New Jersey |
| New Mexico |
| New York |
| North Carolina |
| Ohio |
| Oklahoma |
| Oregon |
| Pennsylvania |
| Rhode Island |
| South Carolina |
| Tennessee |
| Texas |
| Utah |
| Virginia |
| Washington |
| West Virginia |
| Wisconsin |

Note: Decedents from 44 states inclusive of the District of Columbia were included in the analysis. Due to NVDRS program privacy rules about small cell sizes and preventing the potential identification of decedents included in the NVDRS, we are unable to provide the number of decedents per state. Notably, as shown in Table 1 and Appendix Table A2, decedents from all U.S. Census Regions were included in the main analyses, which was geographically representative of all Black adult firearm suicide decedents included in the NVDRS over the study period.

Appendix Table A2. Describing the study sample compared to all Black adult firearm suicide decedents documented in the NVDRS, 2013-2021.

|  | Study sample (n = 843) | |  | All decedents (n = 8,434) | |  |
| --- | --- | --- | --- | --- | --- | --- |
|  | Mean/ Frequency | Standard Deviation/Percent |  | Mean/ Frequency | Standard Deviation/Percent | P value |
| Age in years, mean, SD | 38.2 | 16.4 |  | 38.2 | 16.5 | 0.97 |
| Sex, n, % |  |  |  |  |  | 0.93 |
| Male | 747 | 88.6% |  | 7,482 | 88.7% |  |
| Female | 96 | 11.4% |  | 952 | 11.3% |  |
| Ethnicity, n, % |  |  |  |  |  | 0.98 |
| Not Hispanic/Latino/a | 823 | 97.6% |  | 8,232 | 97.6% |  |
| Hispanic/Latino/a | 20 | 2.4% |  | 202 | 2.4% |  |
| Education Level, n, % |  |  |  |  |  | 0.96 |
| 8th grade or less | 12 | 1.4% |  | 132 | 1.6% |  |
| 9th to 12th grade, no diploma | 111 | 13.1% |  | 1,094 | 13.0% |  |
| High school graduate or GED completed | 379 | 45.0% |  | 3,785 | 44.9% |  |
| Some college credit, but no degree | 167 | 19.8% |  | 1,593 | 18.9% |  |
| Associate's degree | 58 | 6.9% |  | 540 | 6.4% |  |
| Bachelor's degree | 55 | 6.5% |  | 612 | 7.3% |  |
| Master's degree, Doctorate or Professional degree | 23 | 2.8% |  | 245 | 2.8% |  |
| Unknown | 36 | 4.5% |  | 363 | 5.1% |  |
| Marital Status, n, % |  |  |  |  |  | 0.71 |
| Married/Civil Union/Domestic Partnership | 192 | 22.8% |  | 2,036 | 24.1% |  |
| Never Married | 483 | 57.3% |  | 4,782 | 56.7% |  |
| Widowed | 29 | 3.4% |  | 289 | 3.4% |  |
| Divorced | 90 | 10.7% |  | 936 | 11.1% |  |
| Married/Civil Union/Domestic Partnership, but separated | 21 | 2.5% |  | 191 | 2.2% |  |
| Single, not otherwise specified | 17 | 2.0% |  | 123 | 1.5% |  |
| Unknown | 11 | 1.3% |  | 77 | 1.0% |  |
| Military Veteran Status, n, % |  |  |  |  |  | 0.88 |
| Did not serve | 684 | 81.1% |  | 6,823 | 80.9% |  |
| Served in the U.S. Armed Forces | 142 | 16.9% |  | 1,418 | 16.8% |  |
| Unknown | 17 | 2.0% |  | 193 | 2.3% |  |
| Death Year, n, % |  |  |  |  |  | 0.89 |
| 2013 | 36 | 4.2% |  | 372 | 4.4% |  |
| 2014 | 39 | 4.6% |  | 460 | 5.4% |  |
| 2015 | 49 | 5.8% |  | 524 | 6.2% |  |
| 2016 | 80 | 9.4% |  | 739 | 8.8% |  |
| 2017 | 83 | 9.9% |  | 862 | 10.2% |  |
| 2018 | 117 | 13.9% |  | 1,036 | 12.3% |  |
| 2019 | 108 | 12.8% |  | 1,067 | 12.7% |  |
| 2020 | 144 | 17.1% |  | 1,501 | 17.8% |  |
| 2021 | 187 | 22.3% |  | 1,873 | 22.2% |  |
| Census Region |  |  |  |  |  |  |
| West | 77 | 9.1% |  | 800 | 9.5% | 0.81 |
| Midwest | 221 | 26.2% |  | 2,116 | 25.1% |  |
| Northeast | 78 | 9.3% |  | 736 | 8.7% |  |
| South | 467 | 55.4% |  | 4,782 | 56.7% |  |

Author’s analysis of National Violent Death Reporting System (NVDRS) Restricted Access Database (RAD) data.

Appendix Table A3. List of first-cycle codes.

| **Code** |
| --- |
| Mental health |
| Recreational/illicit substance involved |
| Argument |
| 9mm caliber |
| Alcohol involved |
| Relationship problem |
| Physical health |
| Previous suicidal ideation |
| Health care involved |
| Firearm violence against others |
| Legal issues |
| Advanced warning |
| Children at scene |
| Financial problems |
| Previous suicide attempt |
| .40 caliber |
| Depressed mood/stress |
| Substance use history |
| Treatment non-adherence |
| Loved one loss |
| Medication |
| .380 caliber |
| Expressing love |
| Suicide in front of someone |
| Employment problems |
| .38 caliber |
| Abusive |
| Shotgun |
| Socially isolated |
| Military veteran |
| .45 caliber |
| Paranoia |
| Legally purchased gun |
| Unsafely stored/unlocked gun |
| .22 caliber |
| Gun recently purchased |
| Estranged from family |
| Partner/family member's gun |
| Expressing apology |
| .357 caliber |
| Criminal history |
| Sexual misconduct |
| Financial problems: Housing |
| Pain |
| Multiple guns |
| Disability |
| Family dispute |
| Gun stolen |
| Admitted to treatment facility |
| Car accident |
| Illegal gun |
| Social media |
| Religion/faith |
| Giving gifts |
| On phone |
| Pregnancy |
| Carried gun actively |
| Found by friend |
| Unobtained life/career goals |
| Accused of crime |
| Recipient of violence/trauma |
| Rifle |
| Russian roulette |
| Terminal illness |
| Police officer |
| Safe storage/locked gun |
| Childhood trauma |
| Dispute over child/custody battle |
| Old age |
| Shooting range |
| Attempted gun removal |
| Friend's gun |

Notes: This is a list of the most common first-cycle codes from the analysis of recurring and salient personal circumstance preceding firearm suicide death among Black adults.
